# Evolution of opposing regulatory interactions underlies the emergence of eukaryotic cell cycle checkpoints

**DOI:** 10.1038/s41598-021-90384-3

**Published:** 2021-05-27

**Authors:** Rosa D. Hernansaiz-Ballesteros, Csenge Földi, Luca Cardelli, László G. Nagy, Attila Csikász-Nagy

**Affiliations:** 1 Randall Centre for Cell and Molecular Biophysics, King’s College London, London, SE1 1UL UK; 2 Faculty of Medicine, Institute for Computational Biomedicine, Bioquant, Heidelberg University, 69120 Heidelberg, Germany; 3 Synthetic and Systems Biology Unit, Institute of Biochemistry, Biological Research Centre, Szeged, 6726 Hungary; 4 Department of Computer Science, University of Oxford, Wolfson Building, Parks Road, Oxford, OX1 3QD UK; 5 Faculty of Information Technology and Bionics, Pázmány Péter Catholic University, Práter u. 50/A, Budapest, 1083 Hungary

**Keywords:** Bioinformatics, Cell division, Coevolution, Phylogenetics, Biochemical reaction networks, Differential equations, Dynamical systems, Oscillators, Checkpoints

## Abstract

In eukaryotes the entry into mitosis is initiated by activation of cyclin-dependent kinases (CDKs), which in turn activate a large number of protein kinases to induce all mitotic processes. The general view is that kinases are active in mitosis and phosphatases turn them off in interphase. Kinases activate each other by cross- and self-phosphorylation, while phosphatases remove these phosphate groups to inactivate kinases. Crucial exceptions to this general rule are the interphase kinase Wee1 and the mitotic phosphatase Cdc25. Together they directly control CDK in an opposite way of the general rule of mitotic phosphorylation and interphase dephosphorylation. Here we investigate why this opposite system emerged and got fixed in almost all eukaryotes. Our results show that this reversed action of a kinase-phosphatase pair, Wee1 and Cdc25, on CDK is particularly suited to establish a stable G2 phase and to add checkpoints to the cell cycle. We show that all these regulators appeared together in LECA (Last Eukaryote Common Ancestor) and co-evolved in eukaryotes, suggesting that this twist in kinase-phosphatase regulation was a crucial step happening at the emergence of eukaryotes.

## Introduction

Entry into mitosis—the transition from the second gap phase (G2) to the mitotic (M) phase—is a crucial step in the eukaryotic cell cycle^1–4^. This transition, and indeed the whole cell cycle, is tightly controlled by a regulatory network involving an extensive coordination of phosphorylation and dephosphorylation events, catalyzed by protein kinases and phosphatases^5^. Kinases add phosphate groups to modify the target molecules, while phosphatases remove these modifications. These kinases and phosphatases are also subject to the same types of post-translational modifications^6,7^.

**Figure 1:**
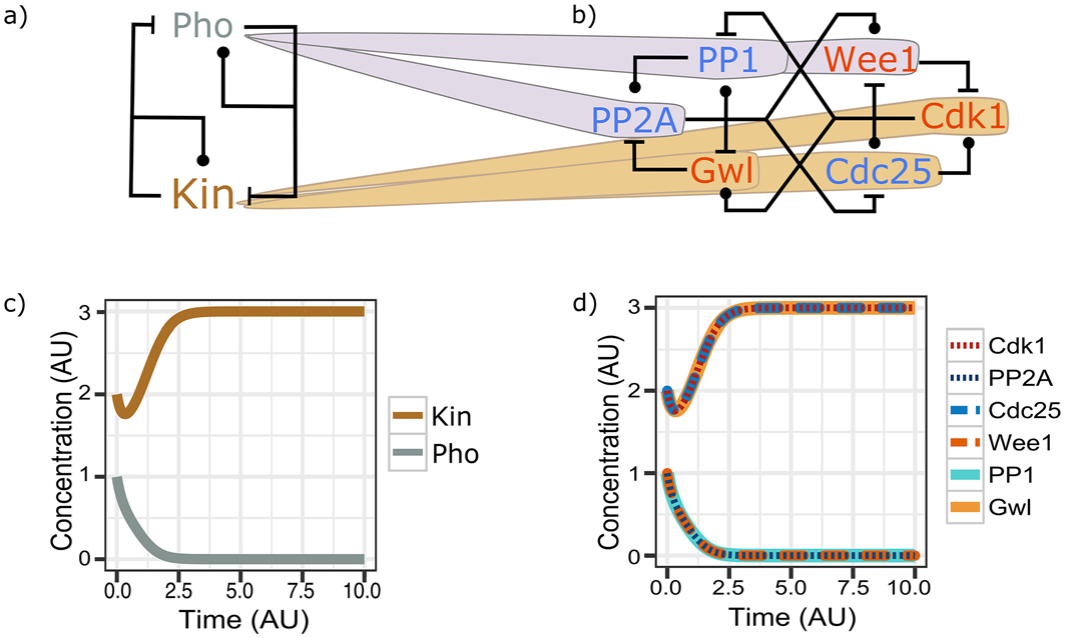
Similar dynamical behaviour of the mitotic entry and the mutual inhibition (MI) systems. (**a**) Wiring diagram of the Mutual Inhibition system (MI system). (**b**) Wiring diagram of the regulatory network of the G2/M transition. Colours of molecular species show their biological type: PP1, PP2 and Cdc25 are phosphatases (blue), and Gwl, Wee1 and Cdk1 are kinases (orange). Edges indicate catalytic reactions: ball-end activation; dash-end inhibition. Both networks are shown as influence networks^36^ (Supplementary Figure S1). Shadows of molecules in the G2M system point to their corresponding species in the MI system. (**c**) Time-course diagram of MI system. (**d**) Time-course diagram of the G2/M system. Diagrams show the active forms from each species. Active forms of Cdk1, Cdc25 and Gwl collapse into the ‘Mitosis’ trace of MI (brown), while active forms of PP2A, PP1 and Wee1 collapse into the ‘Interphase’ trace of MI (grey). See supplementary methods for details of the simulations. Graphics were obtained by R version 3.6 and ggplot2^37,38^.

The regulatory network controlling mitotic onset has been described as a tug-of-war between kinases and phosphatases^8^. Phosphatases fight to keep the system in interphase, while kinases push progression into mitosis^9–11^. Cyclin-dependent kinases are the main controllers of entry into mitosis, while exit from mitosis is induced by Aurora kinase^12^, Polo kinase^13^ and other mitotic kinases. However, there are exceptions. During mitotic onset, the biochemical functions of some of the key molecules do not match with their biological function. Specifically, not all phosphatases are interphase factors, and not all kinases promote the entry into mitosis (Fig. 1).

## Molecular network controlling mitotic entry

The core of the regulatory network controlling mitotic entry consists of six molecular species, three kinases and three phosphatases (Fig. 1b)^5,8^. Because these species are highly conserved among eukaryotic organisms, we do not aim to model a specific organism. The main driver of the transition is the kinase Cdk1^14,15^. Cdk1 is phosphorylated and inactivated by the kinase family of Wee1^16^. The phosphatase Cdc25 removes these inhibitory phosphorylations and activates Cdk1^17^. At the same time, Cdk1 controls its regulators via phosphorylation. Cdk1 phosphorylates and activates its activator Cdc25, and inhibits its inhibitor Wee1. Thus, these key players are engaged in two positive feedback loops, one double-negative (Cdk1—|Wee1—|Cdk1) and one pure positive (Cdk1 -> Cdc25 -> Cdk1)^8,18,19^**.**

Wee1 is not the only inhibitor of Cdk1 activity. The PP2A family of phosphatases also indirectly counteracts the activation of Cdk1^20^ by keeping the phosphatase Cdc25 in the dephosphorylated, low activity state^21^. In a similar way, PP2A dephosphorylates and activates the main inhibitor, Wee1, of Cdk1^22^. Cdk1 opposes this indirect inhibition by phosphorylating the key regulators of PP2A: the kinase Greatwall (Gwl) and the phosphatase PP1^23–26^. Cdk1 phosphorylates and activates Gwl, which inhibits PP2A via phosphorylation of ENSA/ARPP-19^27,28^. Cdk1 also phosphorylates PP1, an event that leads to inhibition of the main activator of PP2A^29^.

PP2A controls its regulators by dephosphorylation^30,31^. PP2A dephosphorylates both, PP1 and Gwl, activating the first one and inhibiting the second. Thus, parallel to the Cdk1-Wee1-Cdc25 core, the PP2A-PP1-Gwl core is embedded in two positive feedback loops, one double-negative (PP2A − | Gwl − | PP2A) and one pure positive (PP2A -> PP1 -> PP2A)^5,8,32–34^. As both cores are indirectly regulated, two extra double-negative feedback loops appear, which provides the system with further robustness (Cdk1 -> Gwl − |PP2A -> Wee1 − | Cdk1 and Cdk1 − | PP1 -> PP2A − | Cdc25 -> Cdk1)^35^.

## Results

The dynamic behaviour of the mitotic regulatory network can be emulated by a simpler network^36^, where a single kinase and phosphatase interact (Fig. 1). In this Mutual Inhibition (MI) system the kinase activates itself and inactivates the phosphatase by phosphorylation, while the phosphatase removes these phosphate groups, thereby activating itself and inhibiting the kinase (Fig. 1a).

Mitotic entry can be emulated by the MI system by setting the reaction rates of all interactions equal to 1 (Supplementary Table S1)^36^. Figure 1 shows how the regulatory components of the mitotic system could be grouped based on their dynamical properties. Pho of the MI system (Fig. 1a and Supplementary Figure S2) groups the phosphatases PP2A and PP1 together with the kinase Wee1 (Fig. 1b and Supplementary Figure S3). The dynamical behaviour of these three molecular species overlap with the behaviour of the molecular species Pho of the MI system (Fig. 1c,d). Similarly, Kin in the MI system groups the dynamics of the kinases Cdk1 and Gwl together with the phosphatase Cdc25 (Fig. 1). These two groups of species from the mitotic entry system correspond with the biological functions of a ‘kinase’ that promotes mitosis and a ‘phosphatase’ that promotes interphase, but the biochemical types of Wee1 and Cdc25 are opposite to their corresponding biological functions. The simplified network of mitotic mutual inhibition (MI; Fig. 1a) emphasizes the tug of war between kinases and phosphatases at the transition from phosphatase-dominated interphase to kinase-dominated mitosis. The general framework is that mitotic kinases (respectively, interphase phosphatases) activate each other and inhibit interphase phosphatases (respectively, mitotic kinases), but Cdc25 and Wee1 clearly break this generalized rule.

Cdc25 is not a typical mitotic phosphatase; it has evolved from the rhodanese-like family of phosphatases, far distinct from PP1 and PP2A^17,19,39^. Based on its sequence and structure, Wee1 is classified together with Gwl and Cdk1 in the serine/threonine protein kinase family; however, Wee1 functions as a tyrosine-specific protein kinase^40^. Furthermore, Wee1 and Cdc25 are distinct from other members of the regulatory network of mitotic onset. Typical kinases, such as Gwl and Cdk1, must be phosphorylated on an activation domain to adopt an active conformation^6^. Conversely, typical phosphatases, such as PP1 and PP2A, are active in their dephosphorylated conformation^7,41^. The regulation of Wee1 and Cdc25 does not align with the regulation of these canonical kinases and phosphatases. Contrary to Cdk1 and Gwl, Wee1 does not need to be phosphorylated for activation^40^; rather, Wee1 is inhibited by phosphorylation by Cdk1. Furthermore, Cdc25 requires phosphorylation for its activation, in contrast to other phosphatases, PP2A and PP1, which are active when they are dephosphorylated^42^. The atypical activations of Wee1 and Cdc25 are also reflected in their regulation of Cdk1. Wee1 phosphorylates Cdk1 at an inhibitory site, and the active form of Cdk1 is recovered by dephosphorylation by the phosphatase Cdc25 (Note: Cdk1 has the usual activatory phosphorylation site, phosphorylated by CAK^43^, and Wee1 activity can be increased by phosphorylation on sites other than the Cdk1 target^44^, but these phosphorylations are not involved in the feedback loops we investigate here).

In conclusion, we observe that the biological functions of Wee1 and Cdc25 do not agree with their biochemical functions, and the standard roles of phosphatases and kinases is upside-down in the Cdk1/Wee1/Cdc25 core. It is yet unclear why this atypical regulation of kinases and phosphatases can be observed in the control of a crucial cell cycle transition. In the next section, we investigate the impact of this twist in functions on the dynamical behaviour of the entry into mitosis. Specifically, we compare the dynamical properties of the mitotic entry network with alternative versions of the control system, where the biochemical and biological functions of the regulatory proteins are consistent.

## Cell cycle regulation with atypical kinases and phosphatases

**Figure 2:**
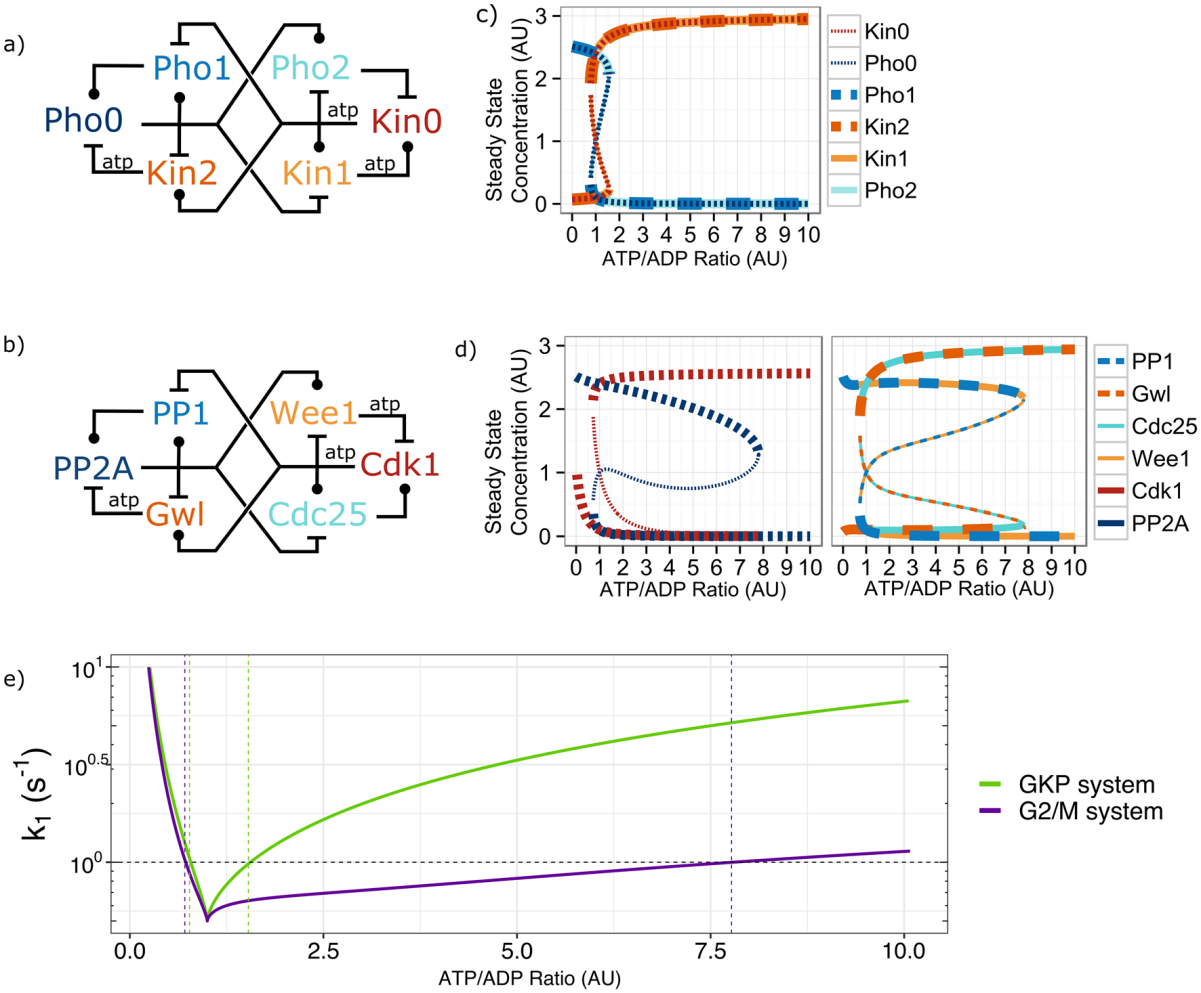
Dynamical analysis of General Kinase-Phosphatase (GKP system) and the mitotic entry network (G2/M system) driven by ATP. (**a**) Wiring diagram of the General Kinase-Phosphatase (GKP). Molecules grouped by biochemical function: blue scale phosphatases; orange scale kinase. Ball-end edges indicate activation; dash-end edges indicate inhibition. (**b**) Wiring diagram of the G2/M regulatory system (notations as on panel a). The atp label indicates the reactions which require these molecules. (**c**) Bifurcation diagram of the GKP system. It represents the steady-state concentration of the noted molecules at various ATP/ADP ratios. The system is bistable in the regime 0.77 < ATP/ADP < 1.53. (**d**) Bifurcation diagrams of the G2/M regulatory system representing the steady-state concentration of the molecules at various ATP/ADP ratios. Stable steady states labelled with thick, unstable steady states with thin dashed lines to allow the visualisation of overlapping curves. The system is bistable in the regime 0.71 < ATP/ADP < 7.77. The left-side panel shows the behaviour of Cdk1 and PP2A, while the right-side panel shows the rest of the species (Gwl, PP1, Wee1 and Cdc25) (**e**) Two-dimensional bifurcation diagram of the GKP and G2/M systems, showing how the bistable region changes as the rate of reactions (k_1_) is changed from the baseline k1 = 1, which was used to plot panels c and d. The vertical dashed lines indicate the bistable region for the GKP and the G2/M systems (green and purple, respectively) at this k_1_ level. See supplementary methods for details on the calculation of these curves. Graphics were obtained using R version 3.6 and ggplot2^37,38^. For panel e, Oscill8^45^ was used.

Figure 1 showed that the biochemical functions of Wee1 and Cdc25 do not match with the mitotic role of typical kinases and phosphatases respectively. To understand the implications of this rewiring on the dynamical behaviour of the mitotic onset, we introduce a model of three kinases and three phosphatases, we term as the General Kinase-Phosphatase (GKP) system (Fig. 2a and Supplementary Figure S4). This system is composed of three copies of interlocked Mutual Inhibition (MI) systems by multiplying components while keeping their biochemical and biological functions in agreement. We are investigating how far the dynamical behaviour of this system differs from that of the real mitotic entry regulatory system with its atypical kinases and phosphatase.

The GKP system shows the same topology as the mitotic entry network (Fig. 2b). However, the kinase Wee1 is replaced by a phosphatase that is inactivating Cdk1, maintaining the coupling between biochemical and biological functions (Pho2 in Fig. 2a). Similarly, the phosphatase Cdc25 is substituted by a kinase and groups with the mitotic factors (Kin1 in Fig. 2a). The other components of the GKP system work exactly as the molecular species of the mitotic entry network (G2/M system, Fig. 2b). Since the GKP system does not exist in nature, we have replaced all the real names by the abbreviation of their biochemical types.

Solely based on the topology of the G2/M network and the GKP system, there should not be a real difference between their dynamical behaviours (Fig. 2a,b). However, we observe a major discrepancy in the way kinases and phosphatases control the (de)phosphorylation cycles^6,7,46^. The function of kinases is tightly linked to the presence of ATP, as these molecules serve as both energy and phosphate source^47–50^. Phosphatases do not demand an energy source for dephosphorylation and use H_2_O molecules as phosphate acceptors to transfer the phosphate group from the phosphoprotein^7,46^.

The cell cycle is a highly energetic demanding process, whose progression is tightly connected with metabolism^51–53^. The oscillations of ATP concentration were proposed to be connected to the cell cycle progression^48,53–56^. In the G1/S phase it is found in its minima; it reaches its peak in late G2 phase, and rapidly drops during mitosis^51,55,57^ The ATP/ADP ratio recently re-emerged as a dynamical driver of the cell cycle, since it is directly controlled by redox reactions and pH^51,53,58–60^. The ATP/ADP ratio clearly controls the rates of phosphorylation reactions^48–50^, but there is less evidence about the direct proportionality of reaction rates to ATP/ADP ratio^61,62^. Furthermore, it has been recently proposed that the switch in the activities of the Cdk1-Cdc25-Wee1 core module can be driven by changes in the ATP/ADP ratio of cells^63^.

Following these lines, we investigate the steady states of both systems (G2/M and GKP) at various levels of ATP/ADP ratio (Fig. 2). The wiring diagrams of Fig. 2a,b show which reactions of the GKP system and the mitotic entry system rely on ATP. For both systems we can see the typical S-shape bifurcation plots of bistable systems^64,65^, since in a given ATP/ADP ratio regime the systems can settle both in a high and a low Kin0 or Cdk1 (Fig. 2c,d respectively) activity states. Similar pictures were drawn for almost all cell cycle transitions, mostly using cell size or abundance of cyclins as external drivers of the system^5,64,66,67^. Here we see that the G2/M system we consider can be driven to mitosis only at a high ATP/ADP ratio (7.77), when the low Cdk1 activity steady state disappears. On the other hand, the GKP system can keep the corresponding Kin0 at a low steady state only up to an ATP/ADP ratio of 1.53 (Fig. 2c,d). This sixfold change difference between the two models is maintained and even more prominent as we change the reaction rates of the two systems parallelly (Fig. 2e). Here we plotted how the bistable region for ATP/ADP ratio changes widens as we increase *k*_1_, the parameter that is controlling the strength of all the enzymatic reactions. When *k*_1_ is small, then background, slow phosphorylation and dephosphorylation reactions maintain a single steady state, but as it increases, the positive feedbacks take over and bistability appears.

The modelled systems of Fig. 2 are certainly simplified versions of the real system. We used equal reaction rates and basic two step modification kinetics in each reaction to simulate a generic model, not specifically fitting to any organism where this network is present (see supplement for details). However, the qualitative characteristics proposed by this analysis, highlights the central role of flipping Wee1 kinase and Cdc25 phosphatase in the G2/M system. The flip could have been essential to create a control on the initiation of mitosis when cells were in an energy rich environment and loaded with ATP. Without this flip, the GKP model cannot provide a wide and stable low kinase activity state, even at low ATP/ADP ratios, below what is naturally observed in eukaryotes^53,61,62,68^.

## Controlling the entry into mitosis

**Figure 3:**
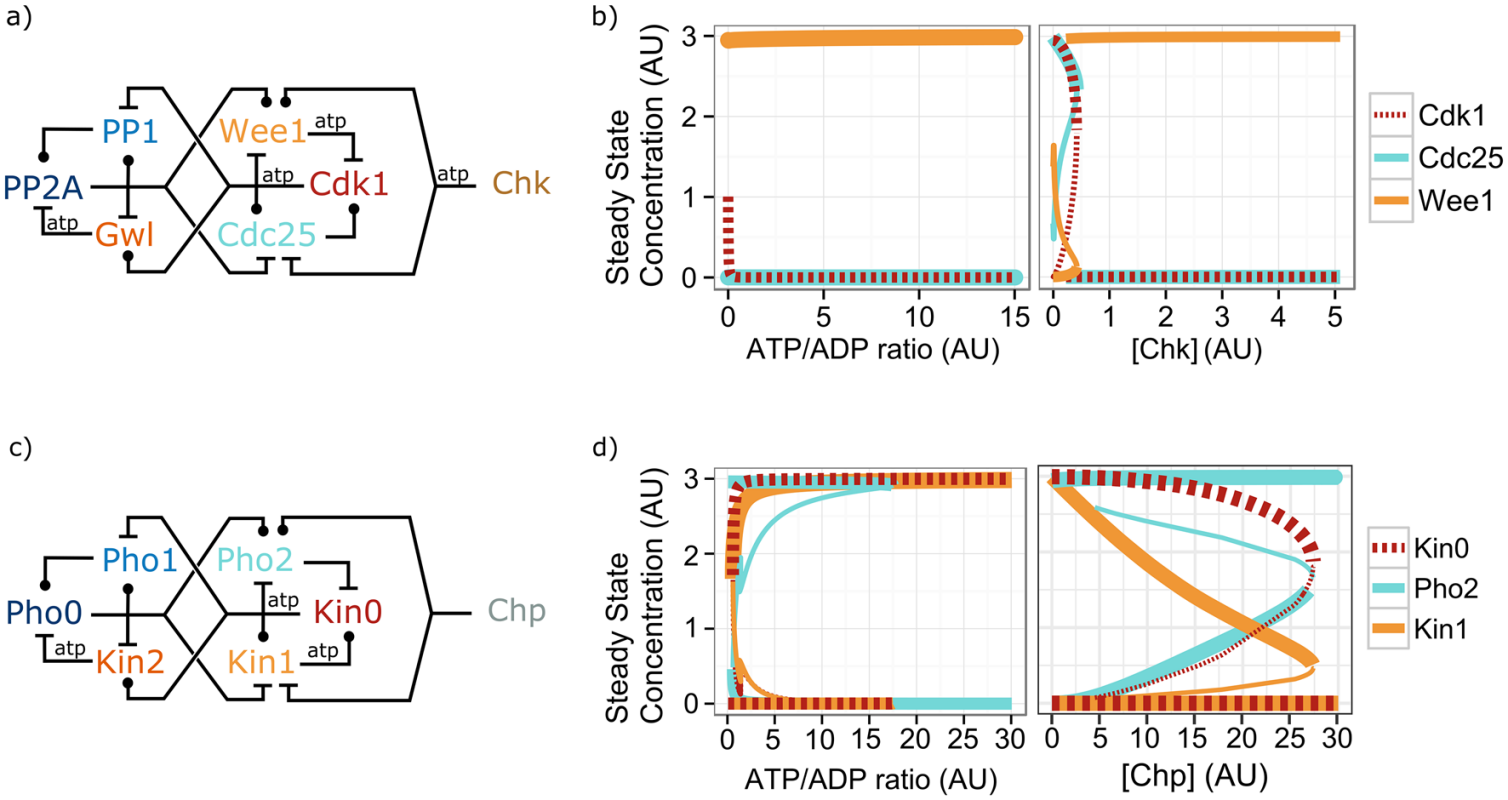
Arresting the entry into mitosis through checkpoint kinases. (**a**) Wiring diagram of the mitotic network controlled by the checkpoint kinase Chk. Blue palette indicates the phosphatases. Orange palette shows the kinases. Ball-end edges indicate activation; dash-end edges indicate inhibition. *atp* labels the edges that require a source of phosphate. (**b**-left) Bifurcation diagram of the steady-state concentration when the ATP/ADP ratio controls the system but Chk parameter is set at 0.5 (AU) (**b**-right) Bifurcation diagram of the steady-state concentration of the core cell cycle controllers with Chk as an external parameter and the ATP/ADP ratio is set to 8 (AU), which was enough to drive the cells into mitosis in the absence of Chk (Fig. 2). (**c**) Wiring diagram of the GKP system interacting with an external phosphatase through Pho2 and Kin1 (Chp-GKP system). Blue palette indicates the phosphatases. Orange palette labels the kinases. Ball-end edges indicate activation; dash-end edges indicate inhibition. *atp* labels the edges that require a source of phosphate. (**d**-left) Bifurcation diagram of the steady-state concentration when the ATP/ADP ratio drives the system and the Chp parameter is set at 0.5 (AU). (**d**-right) Bifurcation diagram of the steady-state concentration when Chp controls the system and the ATP/ADP ratio is set to 8 (AU). See supplementary methods for details of the simulations. R version 3.6 and ggplot2^37,38^ was used to create the plots.

Mitotic entry requires not only the presence of enough ATP in the cells, critical checkpoints ensure that mitosis should start only if the DNA is in a proper condition for mitosis. Any DNA damage needs to be repaired in the G2 phase before the cells enter into mitosis, so cells need to be able to stop mitotic entry upon such damages^69–71^. G2 arrest upon DNA damage is achieved through the activation of the widely conserved ATM-dependent checkpoint kinases (Chk1 and Chk2)^69,70^. When DNA damage is detected, ATM phosphorylates and activates both Chk1 and Chk2 and in their active form, the checkpoint kinases phosphorylate both Wee1 and Cdc25^71–74^. In this case, the phosphorylation of the kinase Wee1 leads to an increase in its activity (or reduced degradation), and the phosphorylation of the phosphatase Cdc25 reduces its activity (or induces its removal). Simplified forms of these interactions were incorporated in a model presented on Fig. 3a and detailed on d Supplementary Figure S5. This system cannot be driven into mitosis by high ATP levels when a small amount of Chk is present (Fig. 3b), still a minimal amount of ATP is needed to bring Cdk1 to 0 level, since Chk also requires some ATP. On Fig. 3b we plot how the level of Chk affects the steady states of the Cdk1 module in the presence of high ATP levels. It is clearly observable that Cdk1 and Cdc25 activities go down to zero and Wee1 activity to maximum already at a low level of Chk. A small bistable region exists, where Cdk1 can be either active or inactive, but this disappears as Chk levels increase above 0.45 AU.

Remarkably, the checkpoint kinases regulate Wee1 and Cdc25 as if they were typical kinase and phosphatase^72–74^. The phosphorylation event activates the kinase Wee1 and inhibits the phosphatase Cdc25. These interactions do not add any extra twists, keeping the Wee1–Cdc25–Cdk1 core as the unique atypical regulation inside the regulatory network of the mitotic entry and possibly suggesting that the regulation of Wee1 and Cdc25 by Chk might be more ancient than the twisted effects of Cdk1. It is worth noting that the Wee1–Cdc25 network is not essential for eukaryotes, but cells missing these molecules are more sensitive to DNA damage^75,76^.

This efficient and simple mechanism of arresting the G2/M transition seems to rely on the upside-down regulation of the Cdk1–Cdc25–Wee1 core. To investigate the effect of this atypical regulation in the blocking of mitotic entry, the GKP system is investigated under the same control by a checkpoint (Fig. 3). In the GKP system phosphatases keep the cells in interphase and kinases drive mitosis. To preserve this property, the checkpoint is controlled by a checkpoint phosphatase (Chp on Fig. 3c and Supplementary Figure S6). This phosphatase Chp controls Kin0 (Cdk1) through the activation of the inhibitor Pho2 (Wee1), and inhibition of the activator Kin1 (Cdc25).

The GKP system affected by the checkpoint phosphatase Chp shows a bistable response both for changes in ATP and Chp levels (Fig. 3). The phosphatase active state can be stabilised only up to a given ATP/ADP ratio, above which kinases eventually overtake and win (Fig. 3d). The increase in Chp level can lead to a stable state with kinases losing, but a kinase winning steady state could still exist until large Chp levels (Fig. 3d). Thus, Chp can increase the threshold where kinases can take over but cannot stabilise the phosphatase winning state for high ATP/ADP ratios. Similar to the checkpoint kinase Chk in the mitotic system (Fig. 3a), the checkpoint phosphatase Chp counteracts the antagonistic feedback loop between Kin0 (Cdk1) and its inhibitor Pho2 (Wee1), and the pure positive loop between Kin0 (Cdk1) and its activator Kin1 (Cdc25) (Fig. 3c). However, high ATP levels can overtake this effect in the GKP system, while in the real mitotic entry network ATP alone cannot drive cells with DNA damage into mitosis. In summary, a system such as the GKP, where all kinases and phosphatases are regulated according to their biochemical function, cannot block the entry into mitosis. Presumably evolution would select against this type of systems, when cells are in an environment highly enriched in energy sources. In contrast, the widely observed atypical regulation of the Cdk1–Cdc25–Wee1 core can induce a stable arrest in G2 phase, which would be selected for.

## Evolutionary perspective on the role of the kinase-phosphatase switch in the emergence of eukaryotes

In the previous sections, we have shown that the twisted regulation of Wee1 and Cdc25 plays a major role in the DNA damage-controlled entry into mitosis. They are not only regulated in the opposite way than the rest of kinases and phosphatases, but they also control CDK in an unorthodox way. The topology of the regulatory network, together with this upside-down regulation, creates an efficient and simple mechanism to arrest the cell cycle in the G2 phase. Here we investigate how this network emerged during the evolution of eukaryotes and evolved together thereafter.

The commitment into mitosis is a highly energy demanding process. During the G2 phase, ATP is accumulated to be used later in mitosis^55^. Early events of the mitotic onset, such as chromosome condensation or spindle assembly^1,8^ need a substantial amount of ATP. However, the events that take place later in anaphase are the ones that consume most of the stored energy^55^. Thus, a system to keep track of the available energy evolved early during evolution^77^. In eukaryotic organisms, this relies on the AMP-activated protein kinase (AMPK)^78–80^, which controls several energy dependent processes, including cell cycle progression^79^. Early eukaryotic cell cycle control systems might have evolved in a way that they allowed cells to enter into mitosis only if cellular energy level reached a critical threshold^81^. Another, an even more ancient system, where energy demand can drive oscillations is the cyanobacterial circadian clock^82–85^. Processes that require high levels of energy are active over the day phase, and the low energy processes occur during the night phase. In many organisms the cell cycle and the circadian clock are coupled^86^, and DNA damage is controlling and further coupling both of them^87^, so there is a good chance that the circadian clock played a crucial role in ancient metabolic control of cell cycle regulation. Apart from timekeepers, primitive biological systems may also hold a mechanism for the control of their cell division. We have shown before^81^ that an antagonistic system of a single kinase-phosphatase pair (MI) is enough to sense energy level and allow kinase activation only above a critical level. In this framework, it is feasible to presume that early kinases may acquire the function of mitotic factors. When the energy source piled up, the kinases increased their activity, promoting the division through the phosphorylation of their substrates.

**Figure 4:**
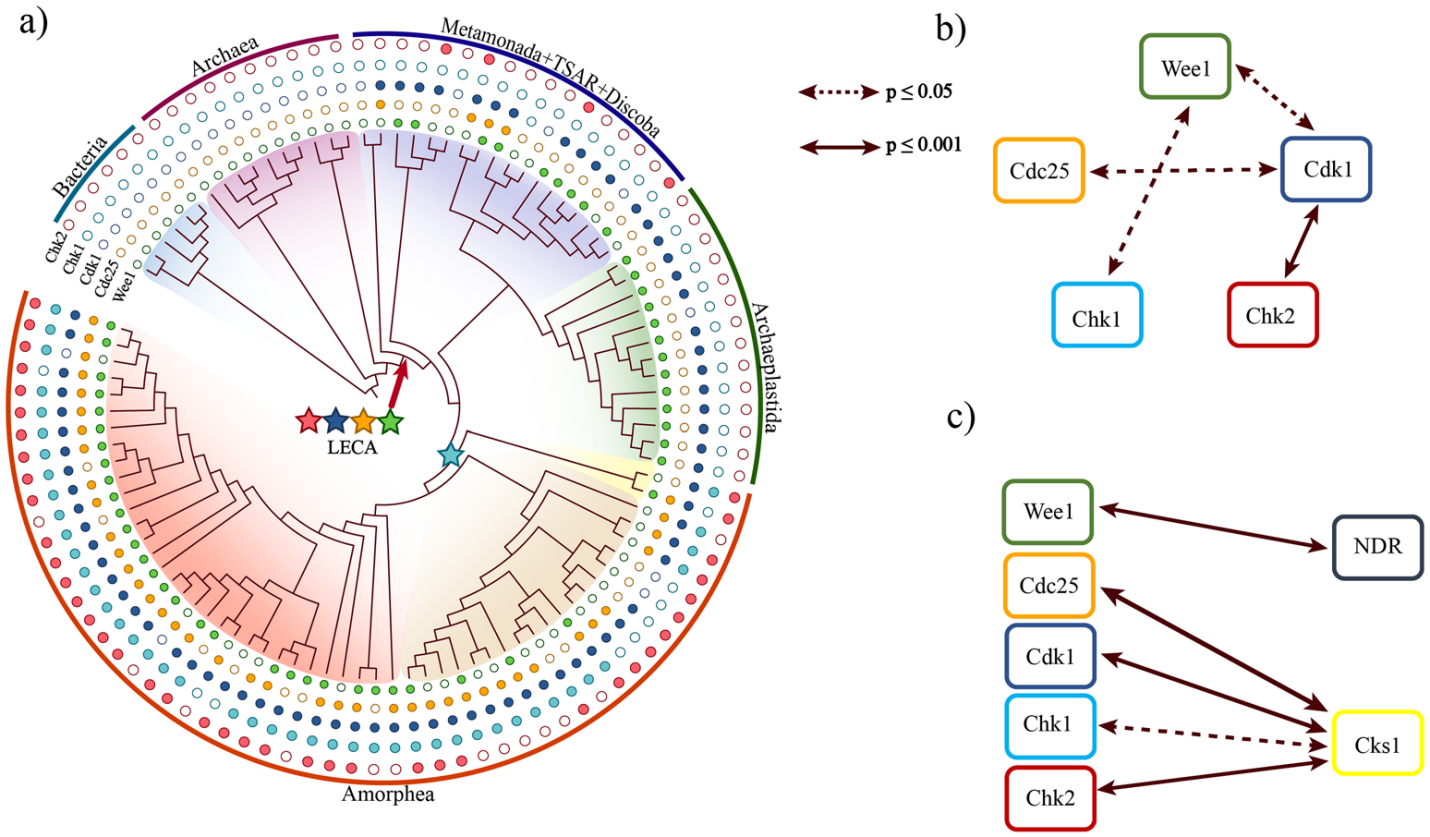
Phylogenetic analyses of Wee1, Cdc25, Cdk1, Chk1 and Chk2. (**a**) Phylogenetic tree (424 species pruned to 100 species for clarity. See the complete phylogenetic tree in Supplementary Fig. S2) with the presence and absence of Chk2, Chk1, Cdk1, Cdc25 and Wee1. Wee1, Cdc25, Cdk1 and Chk2 were present in the last eukaryotic common ancestor (LECA, red arrow), and they were lost several times. Chk1 emerged in the common ancestor of Amorphea, based on ancestral state reconstruction (ASR) analysis. (**b**) Correlated evolution between Wee1, Cdc25, Cdk1, Chk1 and Chk2. Based on the Likelihood ratio test (LRT) results, Wee1 shows correlated evolution with Cdk1 and Chk1 significantly (≤ 0.05, LRT). Cdk1 shows correlated evolution with Cdc25 significantly (*p* ≤ 0.05, LRT) and Chk2 with high significance (*p* ≤ 0.001, LRT). (**c**) Patterns of evolutionary correlation between key kinases as well as proteins chosen as positive (Cks1) and negative controls (NDR). Wee1 shows correlated evolution with NDR (*p* ≤ 0.001, LRT), Cks1 shows correlated evolution with Cdc25, Cdk1, Chk2 (*p* ≤ 0.001, LRT), and with Chk1 (*p* ≤ 0.05, LRT).

The ancient network that regulated cell division in the FECA (first eukaryotic common ancestor), certainly did not contain all factors involved in the current mitotic system. However, some of the key cell division regulators emerged before the appearance of LECA (last eukaryotic common ancestor^88,89^). Since primitive kinases took the function of mitotic factors, molecules that counteract their effect could have taken the opposite role. These last ones could have been the heirs of current phosphatases. Thus, just a primitive group of kinases and phosphatases may have controlled the earliest processes of eukaryotic cell division. Through phylogenetic analyses of correlated evolution, we have investigated Wee1, Cdc25, Cdk1, Chk1 and Chk2 genes (Fig. 4a,b, see also Supplementary Fig. S7, Supplementary Table 2). Ancestral state reconstructions suggest that Wee1, Cdc25, Cdk1 and Chk2 were already present in LECA, and Chk1 appeared in the Amorphea (which includes Opisthokonta and Evosea). That is, of the two checkpoint kinases, Chk2 might be ancient and Chk1 emerged much later afterwards, although it should be noted that denser sampling of early eukaryotes might provide a higher resolution view on the sequence of emergence of these genes. They remained conserved in most eukaryotes (Supplementary Fig. S2), except in the lineage leading to Archaeplastida (the group including plants), where we inferred losses of Cdc25 and Chk2 (Supplementary Fig. S7). It has been postulated that the function of Cdc25 was replaced by CDKs and B-type cyclins^70,71^ and that DNA damage checkpoints use an alternative pathway to stop the cell cycle in plants^87,90^. Indeed, the loss of these genes could be responsible for an increased sensitivity of plants to DNA damage^91^.

We used the likelihood ratio test (LRT) based on Pagel^92^ and Barker et al.^93^ to investigate correlated evolution between the genes above. We inferred that the cell cycle regulator Cdk1 show correlated evolution with Wee1 (*p* ≤ 0.05, LRT), Cdc25 (*p* ≤ 0.05, LRT) and Chk2 (*p* ≤ 0.001, LRT), and that Wee1 show correlated evolution with Chk1 (*p* ≤ 0.05, LRT) (Fig. 4b). We also investigated the co-evolution of these cell cycle G2/M transition regulators with Cks1 and NDR. Cks1 has principal roles in cell cycle regulation as an essential, highly conserved binding partner of CDKs^94^. NDR is a conserved Nuclear Dbf2-Related kinase, which carries out an essential function in late mitosis^95^. Since Cks1 is always present in complex with CDK^96^, this can serve as a positive control for proteins co-evolving with Cdk1. NDR is also a conserved protein kinase, but not in direct connection with CDKs and a role in G2/M transition, so this can serve as a negative control, as a cell cycle kinase that is not expected to closely co-evolve with Cdk1. Our analysis shows that four of the examined proteins (Cdc25, Cdk1, Chk1 and Chk2) showed a significant correlation with Cks1 and only Wee1 showed a correlation with NDR (Fig. 4c).

These analyses show that the Cdk1–Wee1–Cdc25–Chk2 network appeared and co-evolved together to provide a stable G2/M checkpoint for eukaryotes. This finding further supports the claim that the twist in kinase-phosphatase activities at the G2/M transition regulation was a key evolutionary step to create a checkpoint that can stop the eukaryotic cell cycle in case of ongoing DNA replication problem or DNA damage.

## Discussion

Primitive cell cycle regulatory processes might have evolved somewhere between the first and the last common ancestor of eukaryotes to ensure the once-and-only once replication of multiple chromosomes and the accurate partitioning of sister chromatids to the incipient daughter cells. Of equal importance, chromosome replication had to be coordinated with overall cell growth, chromosome replication had to be blocked if the genome were damaged in any way, and mitosis had to be delayed until the cell had acquired sufficient energy stores to complete the process. In particular, cells evolved a system that stops progression through the cell cycle when something goes wrong with the genetic material. But when high levels of ATP push all kinases to be active, and thereby induce cell division, it is challenging to stop cell-cycle progression even if DNA is damaged. The observed flip in the roles of Wee1 kinase and Cdc25 phosphatase could have been sufficient to deal with this challenge. The kinase Wee1 lined up with interphase factors, while the phosphatase Cdc25 teamed up with mitotic factors. In this way, cell division can be arrested in case of any damage to the DNA or if DNA replication is still ongoing. The ability to decouple DNA replication and division became necessary only with the emergence of multiple chromosomes in eukaryotes, explaining why all members of the Cdk1–Wee1–Cdc25 network appeared with the emergence of eukaryotes. Wee1 and Cdc25 are unique enzymes. They are not functionally related to other enzymes in the network, but they are cornerstones of the regulatory system. There is certainly a lot left to be investigated about the evolution of the cell cycle regulatory network in eukaryotes^89,97^. We showed here how a twist in the Cdk1–Wee1–Cdc25 system allowed the cell cycle to be effectively arrested in an energy rich environment.

The observed twist from the general rule of kinase-phosphatase activity to meet the demands of a functional checkpoint might not be unique to the regulation of the G2/M transition. Certainly the G1/S transition is similarly controlled by an isoform of Cdc25 phosphatase^98^ and the mitotic checkpoint was also proposed to be influenced by complex interactions of kinases and phosphatases^99^. The above-mentioned regulation of AMPK by energy level also shows that the AMP-activated protein kinase is inhibited by ATP and activated by a phosphatase, which is again a twisted system. There might be several other examples, where such a flip in kinase/phosphatase regulation ensures that a threshold can be set, before a crucial cellular transition could occur. It is tempting to speculate that the observed twist in kinase-phosphatase interaction could be a more general mechanism for establishing checkpoints and adjustable thresholds in cellular signalling.

## Supplementary information

Supplementary Information 1.

Supplementary Information 2.

## Data Availability

The data underlying this article are available in the article and in its online supplementary material.

## References

[1] Alberts B (2017). Part I introduction to the cell: cells and genomes. Mol. Biol. Cell.

[2] Csikász-Nagy A, Palmisano A, Zámborszky J (2011). Molecular network dynamics of cell cycle control: transitions to start and finish. Methods Mol. Biol..

[3] Novak B, Tyson JJ, Gyorffy B, Csikasz-Nagy A (2007). Irreversible cell-cycle transitions are due to systems-level feedback. Nat. Cell Biol..

[4] Tyson JJ, Csikasz-Nagy A, Novak B (2002). The dynamics of cell cycle regulation. BioEssays.

[5] Fisher D, Krasinska L, Coudreuse D, Novak B (2012). Phosphorylation network dynamics in the control of cell cycle transitions. J. Cell Sci..

[6] Endicott JA, Noble MEM, Johnson LN (2012). The structural basis for control of eukaryotic protein kinases. Annu. Rev. Biochem..

[7] Barford D, Das AK, Egloff M-P (1998). The structure and mechanism of protein phosphatases: insights into catalysis and regulation. Annu. Rev. Biophys. Biomol. Struct..

[8] Domingo-Sananes MR, Kapuy O, Hunt T, Novak B (2011). Switches and latches: a biochemical tug-of-war between the kinases and phosphatases that control mitosis. Philos. Trans. R. Soc. B Biol. .ences.

[9] Bollen M, Gerlich DW, Lesage B (2009). Mitotic phosphatases: from entry guards to exit guides. Trends Cell Biol..

[10] Nigg EA (2001). Mitotic kinases as regulators of cell division and its checkpoints. Nat. Rev. Mol. Cell Biol..

[11] Ma HT, Poon RYC (2011). How protein kinases co-ordinate mitosis in animal cells. Biochem. J..

[12] Carmena M, Earnshaw WC (2003). The cellular geography of Aurora kinases. Nat. Rev. Mol. Cell Biol..

[13] Barr FA, Silljé HHW, Nigg EA (2004). Polo-like kinases and the orchestration of cell division. Nat. Rev. Mol. Cell Biol..

[14] Santamaría D (2007). Cdk1 is sufficient to drive the mammalian cell cycle. Nature.

[15] Hochegger H, Takeda S, Hunt T (2008). Cyclin-dependent kinases and cell-cycle transitions: Does one fit all?. Nat. Rev. Mol. Cell Biol..

[16] Parker LL, Atherton-Fessler S, Piwnica-Worms H (1992). p107wee1 is a dual-specificity kinase that phosphorylates p34cdc2 on tyrosine 15. Proc. Natl. Acad. Sci..

[17] Nilsson I, Hoffmann I (2000). Cell cycle regulation by the Cdc25 phosphatase family. Prog. Cell Cycle Res..

[18] Novak B, Tyson JJ (1993). Numerical analysis of a comprehensive model of M-phase control in Xenopus oocyte extracts and intact embryos. J. Cell Sci..

[19] Perry JA, Kornbluth S (2007). Cdc25 and Wee1: Analogous opposites?. Cell Div..

[20] Lee TH, Solomon MJ, Mumby MC, Kirschner MW (1991). INH, a negative regulator of MPF, is a form of protein phosphatase 2A. Cell.

[21] Clarke PR, Hoffmann I, Draetta G, Karsenti E (1993). Dephosphorylation of cdc25-C by a type-2A protein phosphatase: specific regulation during the cell cycle in Xenopus egg extracts. Mol. Biol. Cell.

[22] Mueller PR, Coleman TR, Dunphy WG (1995). Cell cycle regulation of a Xenopus Wee1-like kinase. Mol. Biol. Cell.

[23] Yu J, Zhao Y, Li Z, Galas S, Goldberg ML (2006). Greatwall kinase participates in the Cdc2 autoregulatory loop in Xenopus egg extracts. Mol. Cell.

[24] Hara M (2012). Greatwall kinase and cyclin B-Cdk1 are both critical constituents of M-phase-promoting factor. Nat. Commun..

[25] Dohadwala M (1994). Phosphorylation and inactivation of protein phosphatase 1 by cyclin-dependent kinases. Proc. Natl. Acad. Sci. U. S. A..

[26] Yamano H, Ishii K, Yanagida M (1994). Phosphorylation of dis2 protein phosphatase at the C-terminal cdc2 consensus and its potential role in cell cycle regulation. EMBO J..

[27] Gharbi-Ayachi A (2010). The substrate of Greatwall kinase, Arpp19, controls mitosis by inhibiting protein phosphatase 2A. Science.

[28] Mochida S, Maslen SL, Skehel M, Hunt T (2010). Greatwall phosphorylates an inhibitor of protein phosphatase 2A that is essential for mitosis. Science.

[29] Hégarat N (2014). PP2A/B55 and Fcp1 regulate Greatwall and Ensa dephosphorylation during mitotic exit. PLoS Genet..

[30] Williams BC (2014). Greatwall-phosphorylated Endosulfine is both an inhibitor and a substrate of PP2A-B55 heterotrimers. Elife.

[31] Grallert A (2015). A PP1–PP2A phosphatase relay controls mitotic progression. Nature.

[32] Hégarat N, Rata S, Hochegger H (2016). Bistability of mitotic entry and exit switches during open mitosis in mammalian cells. BioEssays.

[33] O’Farrell PH (2001). Triggering the all-or-nothing switch into mitosis. Trends Cell Biol..

[34] Tuck C, Zhang T, Potapova T, Malumbres M, Novák B (2013). Robust mitotic entry is ensured by a latching switch. Biol. Open.

[35] Cardelli L, Csikász-Nagy A, Dalchau N, Tribastone M, Tschaikowski M (2016). Noise reduction in complex biological switches. Sci. Rep..

[36] Cardelli L (2014). Morphisms of reaction networks that couple structure to function. BMC Syst. Biol..

[37] R Core Team. R: A language and environment for statistical computing. R Foundation for Statistical Computing, Vienna, Austria. http://www.R-project.org/ (2013).

[38] Wickham, H. ggplot2. *Wiley Interdiscip. Rev. Comput. Stat.* **3**, 180–185 (2011).

[39] Bordo D, Bork P (2002). The rhodanese/Cdc25 phosphatase superfamily. EMBO Rep..

[40] Squire CJ, Dickson JM, Ivanovic I, Baker EN (2005). Structure and inhibition of the human cell cycle checkpoint kinase, Wee1A kinase. Structure.

[41] Seshacharyulu P, Pandey P, Datta K, Batra SK (2013). Phosphatase: PP2A structural importance, regulation and its aberrant expression in cancer. Cancer Lett..

[42] Rudolph J (2007). Cdc25 phosphatases: structure, specificity, and mechanism. Biochemistry.

[43] Morgan DO (1995). Principles of CDK regulation. Nature.

[44] Wu L, Russell P (1993). Nim1 kinase promotes mitosis by inactivating Wee1 tyrosine kinase. Nature.

[45] Conrad, E. D. *Bifurcation Analysis and Qualitative Optimization of Models in Molecular Cell Biology with Applications to the Circadian Clock.* (Virginia Tech, 2006).

[46] Moorhead GBG, Trinkle-Mulcahy L, Ulke-Lemée A (2007). Emerging roles of nuclear protein phosphatases. Nat. Rev. Mol. Cell Biol..

[47] Lane N, Martin WF (2012). The origin of membrane bioenergetics. Cell.

[48] Atkinson DE (1968). Energy charge of the adenylate pool as a regulatory parameter. Interaction with feedback modifiers. Biochemistry.

[49] Szymańska P, Kochańczyk M, Miękisz J, Lipniacki T (2015). Effective reaction rates in diffusion-limited phosphorylation-dephosphorylation cycles. Phys. Rev. E Stat. Nonlinear Soft Matter Phys..

[50] Keshwani MM, Harris TK (2008). Kinetic mechanism of fully activated S6K1 protein kinase. J. Biol. Chem..

[51] Moreira JDV (2015). Cell cycle progression is regulated by intertwined redox oscillators. Theor. Biol. Med. Model..

[52] Xiong W (2012). Regulation of the cell cycle via mitochondrial gene expression and energy metabolism in HeLa cells. Acta Biochim. Biophys. Sin..

[53] Roci I, Watrous JD, Lagerborg KA, Jain M, Nilsson R (2020). Mapping metabolic oscillations during cell cycle progression. Cell Cycle.

[54] Huang S, Li F, Zhou JX, Qian H (2017). Processes on the emergent landscapes of biochemical reaction networks and heterogeneous cell population dynamics: differentiation in living matters. J. R. Soc. Interface.

[55] Marcussen M, Larsen PJ (1996). Cell cycle-dependent regulation of cellular ATP concentration, and depolymerization of the interphase microtubular network induced by elevated cellular ATP concentration in whole fibroblasts. Cell Motil. Cytoskelet..

[56] Pederson T (2003). Historical review: an energy reservoir for mitosis, and its productive wake. Trends Biochem. Sci..

[57] Futcher B (2006). Metabolic cycle, cell cycle, and the finishing kick to Start. Genome Biol..

[58] Qian H (2007). Phosphorylation energy hypothesis: open chemical systems and their biological functions. Annu. Rev. Phys. Chem..

[59] Yan Y-B (2016). Creatine kinase in cell cycle regulation and cancer. Amino Acids.

[60] Papagiannakis A, Niebel B, Wit EC, Heinemann M (2017). Autonomous metabolic oscillations robustly gate the early and late cell cycle. Mol. Cell.

[61] Berg J, Hung YP, Yellen G (2009). A genetically encoded fluorescent reporter of ATP:ADP ratio. Nat. Methods.

[62] Erecińska M, Silver IA (1994). Ions and energy in mammalian brain. Prog. Neurobiol..

[63] Wang, T. *et al.* Phosphorylation energy and nonlinear kinetics as key determinants for G2/M transition in fission yeast cell cycle. *arXiv preprint* arxiv:1610.09637, 10.1101/084400 (2016).

[64] Tyson JJ, Chen KC, Novak B (2003). Sniffers, buzzers, toggles and blinkers: dynamics of regulatory and signaling pathways in the cell. Curr. Opin. Cell Biol..

[65] Ingalls, B. P. *Mathematical Modeling in Systems Biology: An Introduction* (MIT Press, 2013).

[66] Pomerening JR, Sontag ED, Ferrell JE (2003). Building a cell cycle oscillator: hysteresis and bistability in the activation of Cdc2. Nat. Cell Biol..

[67] Verdugo A, Vinod PK, Tyson JJ, Novak B (2013). Molecular mechanisms creating bistable switches at cell cycle transitions. Open Biol..

[68] Tantama M, Martínez-François JR, Mongeon R, Yellen G (2013). Imaging energy status in live cells with a fluorescent biosensor of the intracellular ATP-to-ADP ratio. Nat. Commun..

[69] Vermeulen K, Van Bockstaele DR, Berneman ZN (2003). The cell cycle: a review of regulation, deregulation and therapeutic targets in cancer. Cell Prolif..

[70] Reinhardt HC, Christian Reinhardt H, Yaffe MB (2009). Kinases that control the cell cycle in response to DNA damage: Chk1, Chk2, and MK2. Curr. Opin. Cell Biol..

[71] Sanchez Y (1997). Conservation of the Chk1 checkpoint pathway in mammals: linkage of DNA damage to Cdk regulation through Cdc25. Science.

[72] Lee J, Kumagai A, Dunphy WG (2001). Positive regulation of Wee1 by Chk1 and 14-3-3 proteins. Mol. Biol. Cell.

[73] Furnari B (1997). Cdc25 mitotic inducer targeted by Chk1 DNA damage checkpoint kinase. Science.

[74] O’Connell MJ (1997). Chk1 is a wee1 kinase in the G2 DNA damage checkpoint inhibiting cdc2 by Y15 phosphorylation. EMBO J..

[75] Cross FR, Siggia ED (2005). Shake it, don’t break it: positive feedback and the evolution of oscillator design. Dev. Cell.

[76] Coudreuse D, Nurse P (2010). Driving the cell cycle with a minimal CDK control network. Nature.

[77] Roustan V, Jain A, Teige M, Ebersberger I, Weckwerth W (2016). An evolutionary perspective of AMPK-TOR signaling in the three domains of life. J. Exp. Bot..

[78] Hardie DG (2011). AMP-activated protein kinase: an energy sensor that regulates all aspects of cell function. Genes Dev..

[79] Hardie DG (2018). Keeping the home fires burning: AMP-activated protein kinase. J. R. Soc. Interface.

[80] Oakhill JS, Scott JW, Kemp BE (2012). AMPK functions as an adenylate charge-regulated protein kinase. Trends Endocrinol. Metab..

[81] Hernansaiz-Ballesteros RD, Cardelli L, Csikász-Nagy A (2018). Single molecules can operate as primitive biological sensors, switches and oscillators. BMC Syst. Biol..

[82] Nakajima M (2005). Reconstitution of circadian oscillation of cyanobacterial KaiC phosphorylation in vitro. Science.

[83] Simons MJP (2009). The evolution of the cyanobacterial posttranslational clock from a primitive ‘phoscillator’. J. Biol. Rhythms.

[84] Kageyama H (2006). Cyanobacterial circadian pacemaker: Kai protein complex dynamics in the KaiC phosphorylation cycle in vitro. Mol. Cell.

[85] Terauchi K (2007). ATPase activity of KaiC determines the basic timing for circadian clock of cyanobacteria. Proc. Natl. Acad. Sci..

[86] Tandem R (2007). Circadian clocks and the cell cycle. Cell.

[87] Chen Z, McKnight SL (2007). A conserved DNA damage response pathway responsible for coupling the cell division cycle to the circadian and metabolic cycles. Cell Cycle.

[88] Cao L (2014). Phylogenetic analysis of CDK and cyclin proteins in premetazoan lineages. BMC Evol. Biol..

[89] Harashima H, Dissmeyer N, Schnittger A (2013). Cell cycle control across the eukaryotic kingdom. Trends Cell Biol..

[90] Dissmeyer N (2009). Control of cell proliferation, organ growth, and DNA damage response operate independently of dephosphorylation of the Arabidopsis Cdk1 homolog CDKA;1. Plant Cell.

[91] Francis D (2011). A commentary on the G2/M transition of the plant cell cycle. Ann. Bot..

[92] Pagel, M. Detecting correlated evolution on phylogenies: a general method for the comparative analysis of discrete characters. *Proc. R. Soc. Lond. B Biol. Sci.* **255**, 37–45 (1994).

[93] Barker D, Meade A, Pagel M (2007). Constrained models of evolution lead to improved prediction of functional linkage from correlated gain and loss of genes. Bioinformatics.

[94] Brown NR (2015). CDK1 structures reveal conserved and unique features of the essential cell cycle CDK. Nat. Commun..

[95] Hergovich A, Stegert MR, Schmitz D, Hemmings BA (2006). NDR kinases regulate essential cell processes from yeast to humans. Nat. Rev. Mol. Cell Biol..

[96] Reynard GJ, Reynolds W, Verma R, Deshaies RJ (2000). Cks1 is required for G_1_ cyclin–cyclin-dependent kinase activity in budding yeast. Mol. Cell. Biol..

[97] Cross FR, Buchler NE, Skotheim JM (2011). Evolution of networks and sequences in eukaryotic cell cycle control. Philos. Trans. R. Soc. B Biol. Sci..

[98] Hoffmann I, Draetta G, Karsenti E (1994). Activation of the phosphatase activity of human cdc25A by a cdk2-cyclin E dependent phosphorylation at the G1/S transition. EMBO J..

[99] Saurin AT (2018). Kinase and phosphatase cross-talk at the kinetochore. Front. Cell Dev. Biol..

