# Supplementary material for: Evolution of opposing regulatory interactions underlies the emergence of eukaryotic cell cycle checkpoints
Source: Sci Rep. 2021 May 27;11:11122. doi: 10.1038/s41598-021-90384-3 (PMC8159995; doi:10.1038/s41598-021-90384-3)
Supplement: Supplementary file 2 — Supplementary Information 2. [file 41598_2021_90384_MOESM2_ESM.pdf]

Evolution of opposing regulatory interactions underlies the emergence of eukaryotic cell cycle checkpoints

Rosa D. Hernansaiz-Ballesteros[1,2], Csenge Földi[3], Luca Cardelli[4], László G. Nagy[3] & Attila Csikász-Nagy[1,5]

[1] Randall Centre for Cell and Molecular Biophysics, King's College London, SE1 1UL London, UK

[2] Institute for Computational Biomedicine, Heidelberg University, Faculty of Medicine, Bioquant, 69120 Heidelberg, Germany

[3] Synthetic and Systems Biology Unit, Institute of Biochemistry, Biological Research Centre, Szeged 6726, Hungary

[4] Department of Computer Science, University of Oxford, Wolfson Building, Parks Road, Oxford OX1 3QD, UK

[5] Faculty of Information Technology and Bionics, Pázmány Péter Catholic University, H-1083 Budapest, Hungary

SUPPLEMENTARY METHODS

Modelling Framework

1. **Influence networks and general assumptions**

The models described in the main text are graphically represented as influence networks [1]. Influence networks precisely represent the interactions between the elements of a biological system. For the models presented here, each protein kinase and phosphatase is represented by a node, while the interactions between nodes are (de)phosphorylation events. A node that represents a protein implies that all possible conformations due to multi-site phosphorylation events are also included within that particular node. Particularly, all the alternative conformations are constrained into only three distinctive conformations: active, intermediate and inactive (Figure S1).

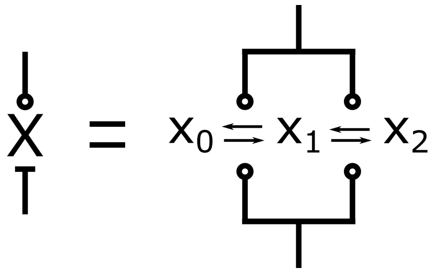

Figure S1. **Notation of triplet motifs.** Left, form that triplet motifs take in an influence network. The influence node X can be regulated through reactions of activation (ball-end) and/or reactions of inhibitions (dash-end). Right, detailed form of the triplet motif. The influence node X can exist in three different conformations: x_0, x_1 and x_2. The reactions of activation move conformations x_2 and x_1 to x_0, while the inhibitory reactions push conformations x_0 and x_1 to x_2.

This approach is a simplification, as theoretically, multi-site proteins regulated through phosphorylation may exist in multiple phosphorylated states, having some of them different functional characteristics [2,3]. Thus, in the models it is assumed that the active conformation is the one that keeps the full activity of the protein; the inactive conformation is the one that does not present any kind of activity; and the intermediate conformation stands for the rest of states that the protein may present. The translation of these constraints into mathematical descriptions is through the use of triplet motifs [1]. A triplet motif refers to a single entity X that can adopt three different states: x_0, x_1 and x_2 (Figure S1). Species X is either a kinase or a phosphatase of an influence network. The x_0 state is assumed as the active conformation, the x_1 as the intermediate, and the x_2 as the inactive conformation. Note that the active conformation is the one that interacts with the species to turn them into their active or inactive conformations.

The use of triplet motifs also indicates the molecular regulatory mechanism assumed for the modelling. This approach explicitly presents a sequential mechanism of (de)phosphorylation. To inhibit a protein X that is found in its active conformation x_0, the order of (de)phosphorylation events first turns this conformation x_0 into the intermediary form x_1, from which the inactive conformation x_2 is reached. The opposite transition path is taken to activate a protein X that is found in its inactive conformation: $x_2 \rightarrow x_1 \rightarrow x_0$. This sequential (de)phosphorylation is based on distributive kinetics, which defines that each single encounter of two different conformations produces at most one modification in one of them.

Kinases and phosphatases are distinct in many biochemical ways. One of their major differences is the (de)phosphorylation mechanism. Kinases require a molecule that works as a source of energy and phosphate donor, while phosphatases just need a phosphate acceptor. The molecule that kinases require for the phosphorylation event is the Adenosine TriPhosphate (ATP); and the phosphatases use a molecule of water for the dephosphorylation. Although the level of ATP is quite stable in a biological system, it locally varies due to its use in many diverse processes [4,5]. This energetic dimension is included in the mathematical descriptions as the *atp* parameter. As *atp* represents the source of energy, this parameter only affects the reactions that are catalysed by kinases.

Regulatory proteins interact with many molecular species. In order to provide an isolated environment to investigate how these molecules interact to produce certain behaviours, the networks are embedded into background reactions. The background reactions mimic the external interactions that a system undergoes. They allow us to understand the dynamical properties of the system without being subjected to the extra interactions that are not relevant for the study. The background reactions are included in the models as opposite fixed biases

pushing towards the different steady states (*hh1* and *hh2* connected through the grey background).

The mathematical systems defined by these premises are constructed to solve the concentrations of active and inactive conformations (x_0 and x_2). The equation that describes the changes in the concentration of the intermediary conformation x_1 is replaced by the subtraction $x_1 = X - x_0 - x_2$, where X indicates the total concentration of the modelled protein. This strategy ensures that the numerical simulations will be within the limits of the desired molecular concentration. Apart from the initial concentrations of the proteins defined by the equations, there are other parameters that are setted. The chosen parameters are a basic set that allows to reproduce the global dynamical behaviour shown in the mitotic onset of the eukaryotic cell cycle. The numerical values used for the main analysis presented in the main text are collected in Table 1.

Supplementary Table 1. **Set of parameters**. Columns indicate the name of the system, and rows show the name of the parameters. ATP level (*atp*), the background fixed bias (*hh1* and *hh2*) and the checkpoint kinase and phosphatase (*chk* and *chp*). *phos* and *kin* are abbreviations that refer to phosphatase and kinase, respectively. The total molecular concentration for all species is 3 (AU). All kinetic constants are set up to 1, unless otherwise stated...

	Mutual Inhibition (MI)	G2 phase to Mitosis (G2M)	General Kin-Phos (GKP)	chk-G2 phase to Mitosis (chk-G2M)	chp-general Kin-Phos (chp-GKP)
atp	1.0	[0-10]	[0-10]	[0-15]	[0-30]
chk	-	-	-	[0-5]	-
chp	-	-	-	-	[0-30]
hh1	0.0	0.5	0.5	0.05	0.05
hh2	0.0	0.5	0.5	0.05	0.05

2. **The Mutual Inhibition (MI) system**

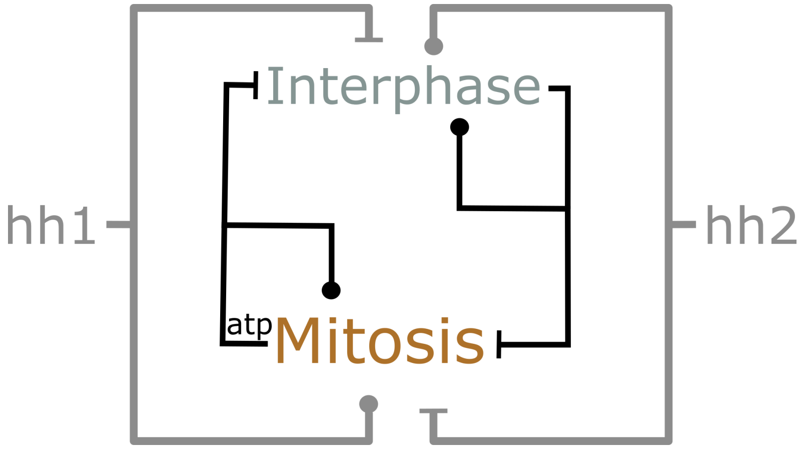

Figure S2. **Mutual Inhibition (MI) system.** The system is composed by two molecular species that counteract each other. Ball-end edges show activation, dash-end edges indicate inhibition. *atp* labels in the edges specify the interactions that are required a source of phosphate and energy. **Initial conditions: Kin (Mitosis)**: z0 = 2, z2 = 1; **Pho (Interphase)**: y0 = 1, y2 = 2.

$$dz0/dt = -y0 \cdot z0 + atp \cdot z0 \cdot (Z - z0 - z2) + hh1 \cdot (Z - z0 - z2) - hh2 \cdot z0,$$

$$dz2/dt = y0 \cdot (Z - z0 - z2) - atp \cdot z0 \cdot z2 - hh1 \cdot z2 + hh2 \cdot (Z - z0 - z2),$$

$$dy0/dt = y0 \cdot (Y - y0 - y2) - atp \cdot z0 \cdot y0 - hh1 \cdot y0 + hh2 \cdot (Y - y0 - y2),$$

$$dy2/dt = -y0 \cdot y2 + atp \cdot z0 \cdot (Y - y0 - y2) + hh1 \cdot (Y - y0 - y2) - hh2 \cdot y2$$

3. The Gap 2 phase to Mitosis (G2M) system

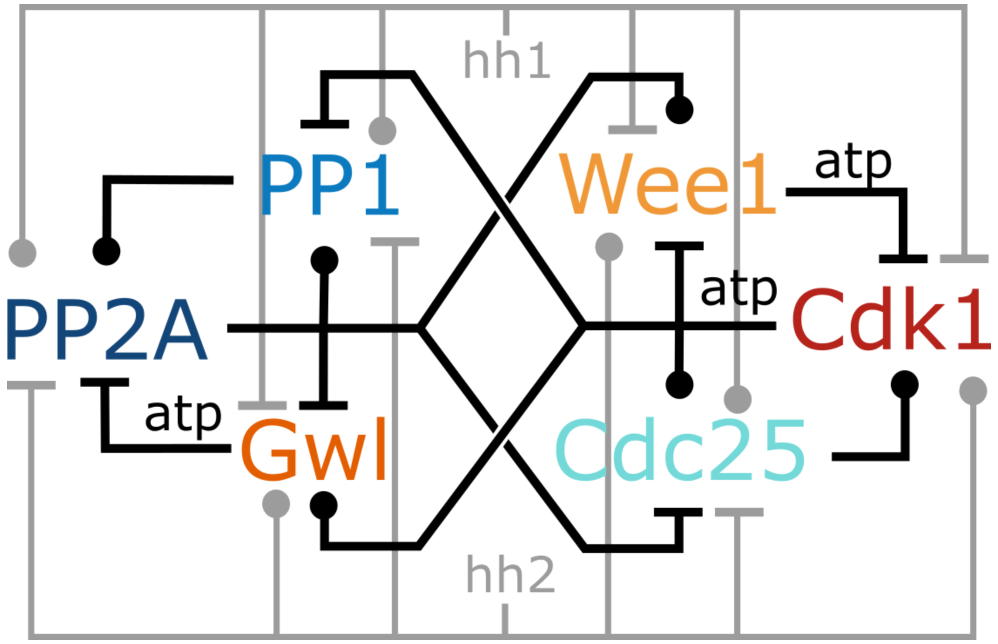

Figure S3. **Regulatory network of the entry into mitosis (G2M system)**. Kinases Cdk1, Gwl and Wee1 (orange palette; Z, P and S, respectively in equations below) and phosphatases PP2A, PP1 and Cdc25 (blue palette; Y, Q and R, respectively in equations below). The background grey wiring indicates the action of the hysteretic harnesses. Ball-end edges show activation, dash-end edges indicate inhibition. *atp* labels in the edges specify the interactions that required a source of phosphate and energy. **Initial conditions for Figure 1: Cdk1**: z0 = 2, z2 = 1; **Gwl**: p0 = 2, p2 = 1; **Wee1**: s0 = 1, s2 = 2; **PP2A**: y0 = 1, y2 = 2; **PP1**: q0 = 1, q2 = 2; **Cdc25**: r0 = 2, r2 = 1. hh1 and hh2 are set up to 0. **Initial conditions for Figure 2: Cdk1:** z0 = 1.8, z2 = 1.2; **Gwl**: p0 = 1.8, p2 = 1.2; **Wee1**: s0 = 1.2, s2 = 1.8; **PP2A**: y0 = 1.2, y2 = 1.8; **PP1**: q0 = 1.2, q2 = 1.8; **Cdc25**: r0 = 1.8, r2 = 1.2. k1 is the kinetic parameter that is used as variable to create Figure 2E.

$$dz0/dt = -k1 \cdot atp \cdot s0 \cdot z0 + k1 \cdot r0 \cdot (Z - z0 - z2) + hh2 \cdot (Z - z0 - z2) - hh1 \cdot z0,$$

$$dz2/dt = k1 \cdot atp \cdot s0 \cdot (Z - z0 - z2) - k1 \cdot r0 \cdot z2 - hh2 \cdot z2 + hh1 \cdot (Z - z0 - z2),$$

$$ds0/dt = k1 \cdot y0 \cdot (S - s0 - s2) - k1 \cdot atp \cdot z0 \cdot s0 + hh2 \cdot (S - s0 - s2) - hh1 \cdot s0,$$

$$ds2/dt = -k1 \cdot y0 \cdot s2 + k1 \cdot atp \cdot z0 \cdot (S - s0 - s2) - hh2 \cdot s2 + hh1 \cdot (S - s0 - s2),$$

$$dr0/dt = k1 \cdot atp \cdot z0 \cdot (R - r0 - r2) - k1 \cdot r0 \cdot y0 - hh2 \cdot r0 + hh1 \cdot (R - r0 - r2),$$

$$dr2/dt = -k1 \cdot atp \cdot z0 \cdot r2 + k1 \cdot y0 \cdot (R - r0 - r2) + hh2 \cdot (R - r0 - r2) - hh1 \cdot r2$$

$$dq0/dt = k1 \cdot y0 \cdot (Q - q0 - q2) - k1 \cdot atp \cdot q0 \cdot z0 - hh2 \cdot q0 + hh1 \cdot (Q - q0 - q2),$$

$$dq2/dt = -k1 \cdot y0 \cdot q2 + k1 \cdot atp \cdot z0 \cdot (Q - q0 - q2) + hh2 \cdot (Q - q0 - q2) - hh1 \cdot q2,$$

$$dp0/dt = k1 \cdot atp \cdot z0 \cdot (P - p0 - p2) - k1 \cdot y0 \cdot p0 + hh2 \cdot (P - p0 - p2) - hh1 \cdot p0,$$

$$dp2/dt = -k1 \cdot atp \cdot z0 \cdot p2 + k1 \cdot y0 \cdot (P - p0 - p2) - hh2 \cdot p2 + hh1 \cdot (P - p0 - p2),$$

$$dy0/dt = k1 \cdot q0 \cdot (Y - y0 - y2) - k1 \cdot atp \cdot p0 \cdot y0 - hh2 \cdot y0 + hh1 \cdot (Y - y0 - y2),$$

$$dy2/dt = - k1 \cdot atp \cdot z0 \cdot r2 + k1 \cdot y0 \cdot (Y - y0 - y2) + hh2 \cdot (Y - y0 - y2) - hh1 \cdot y2$$

4. The General Kinase-Phosphatase (GKP) system

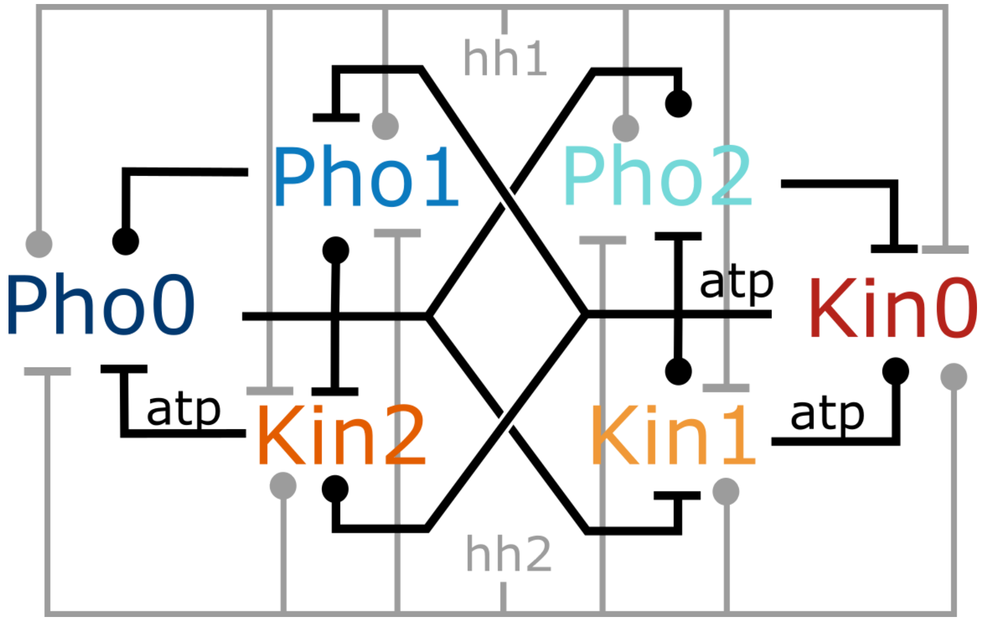

Figure S4. **General Kinase-Phosphatase (GKP) system**. Kinases Kin0, Kin1 and Kin2 (orange palette; Z, R and P, respectively in equations below) and phosphatases Pho0, Pho1 and Pho2 (blue palette; Y, Q and S, respectively in equations below). The background grey wiring indicates the action of the hysteretic harnesses. Ball-end edges show activation, dash-end edges show inhibition. *atp* labels in the edges specify the interactions that required a source of phosphate. **Initial conditions: Kin0**: z0 = 1.8, z2 = 1.2; **Kin1**: r0 = 1.8, r2 = 1.2; **Kin2**: p0 = 1.8, p2 = 1.2; **Pho0**: y0 = 1.2, y2 = 1.8; **Pho1**: q0 = 1.2, q2 = 1.8; **Pho2**: s0 = 1.2, s2 = 1.8. k1 is the kinetic parameter that is used as variable to create Figure 2E.

$$dz0/dt = - k1 \cdot s0 \cdot z0 + k1 \cdot atp \cdot r0 \cdot (Z - z0 - z2) + hh2 \cdot (Z - z0 - z2) - hh1 \cdot z0,$$

$$dz2/dt = k1 \cdot s0 \cdot (Z - z0 - z2) - k1 \cdot atp \cdot r0 \cdot z2 - hh2 \cdot z2 + hh1 \cdot (Z - z0 - z2),$$

$$ds0/dt = k1 \cdot y0 \cdot (S - s0 - s2) - k1 \cdot atp \cdot z0 \cdot s0 + hh2 \cdot (S - s0 - s2) - hh1 \cdot s0,$$

$$ds2/dt = - k1 \cdot y0 \cdot s2 + k1 \cdot atp \cdot z0 \cdot (S - s0 - s2) - hh2 \cdot s2 + hh1 \cdot (S - s0 - s2),$$

$$dr0/dt = k1 \cdot atp \cdot z0 \cdot (R - r0 - r2) - k1 \cdot r0 \cdot y0 - hh2 \cdot r0 + hh1 \cdot (R - r0 - r2),$$

$$dr2/dt = k1 \cdot atp \cdot z0 \cdot r2 + k1 \cdot y0 \cdot (R - r0 - r2) + hh2 \cdot (R - r0 - r2) - hh1 \cdot r2$$

$$dq0/dt = k1 \cdot y0 \cdot (Q - q0 - q2) - k1 \cdot atp \cdot q0 \cdot z0 - hh2 \cdot q0 + hh1 \cdot (Q - q0 - q2),$$

$$dq2/dt = - k1 \cdot y0 \cdot q2 + k1 \cdot atp \cdot z0 \cdot (Q - q0 - q2) + hh2 \cdot (Q - q0 - q2) - hh1 \cdot q2,$$

$$dp0/dt = k1 \cdot atp \cdot z0 \cdot (P - p0 - p2) - k1 \cdot y0 \cdot p0 + hh2 \cdot (P - p0 - p2) - hh1 \cdot p0,$$

$$dp2/dt = - k1 \cdot atp \cdot z0 \cdot p2 + k1 \cdot y0 \cdot (P - p0 - p2) - hh2 \cdot p2 + hh1 \cdot (P - p0 - p2),$$

$$dy0/dt = k1 \cdot q0 \cdot (Y - y0 - y2) - k1 \cdot atp \cdot p0 \cdot y0 - hh2 \cdot y0 + hh1 \cdot (Y - y0 - y2),$$

$$dy2/dt = - k1 \cdot atp \cdot z0 \cdot r2 + k1 \cdot y0 \cdot (Y - y0 - y2) + hh2 \cdot (Y - y0 - y2) - hh1 \cdot y2$$

5. **The Checkpoint-kinase Gap 2 phase to Mitosis (chk-G2M) system**

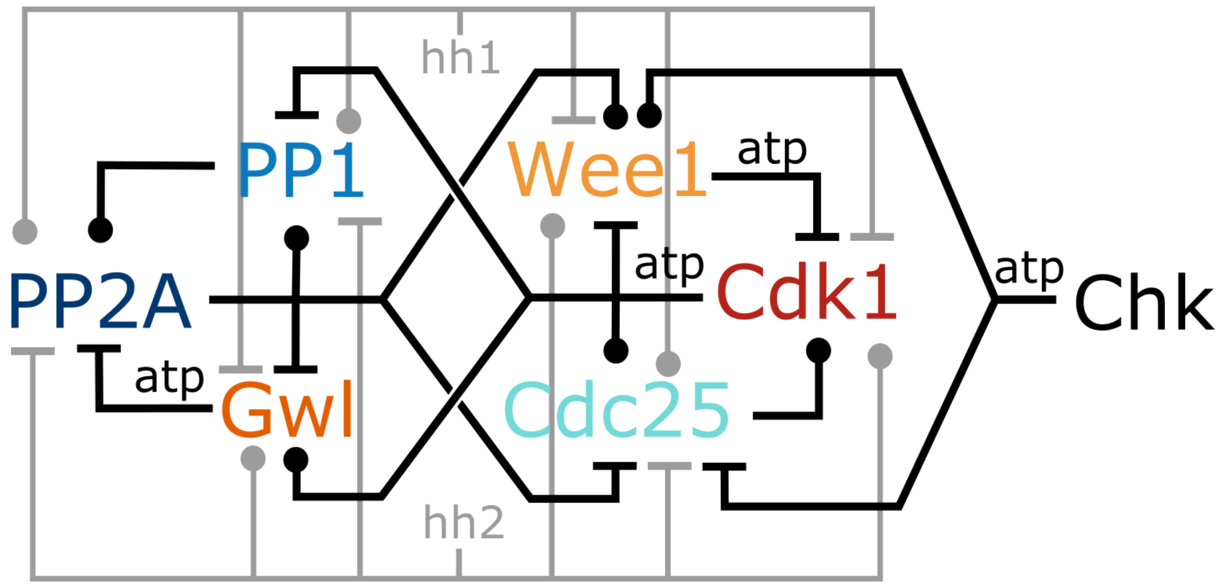

Figure S5. **The Checkpoint-kinase Gap 2 phase to Mitosis (chk-G2M) system**. Chk1 activates the kinase Wee1 and inhibits the phosphatase Cdc25. Kinases Cdk1, Gwl and Wee1 (orange palette; Z, P and S, respectively in equations below) and phosphatases PP2A, PP1 and Cdc25 (blue palette; Y, Q and R, respectively in equations below) are embedded in hysteretic harnesses (grey wiring). Ball-end edges show activation, dash-end edges show inhibition. *atp* labels in the edges specify the interactions that require a source of phosphate. Chk1 is a kinase, so it is also affected by the ATP/ADP ratio. **Initial conditions: Cdk1**: z0 = 1.8, z2 = 1.2; **Gwl**: p0 = 1.8, p2 = 1.2; **Wee1**: s0 = 1.2, s2 = 1.8; **PP2A**: y0 = 1.2, y2 = 1.8; **PP1**: q0 = 1.2, q2 = 1.8; **Cdc25**: r0 = 1.8, r2 = 1.2.

$$dz0/dt = - atp \cdot s0 \cdot z0 + r0 \cdot (Z - z0 - z2) + hh2 \cdot (Z - z0 - z2) - hh1 \cdot z0,$$

$$dz2/dt = atp \cdot s0 \cdot (Z - z0 - z2) - r0 \cdot z2 - hh2 \cdot z2 + hh1 \cdot (Z - z0 - z2),$$

$$ds0/dt = y0 \cdot (S - s0 - s2) - atp \cdot z0 \cdot s0 + hh2 \cdot (S - s0 - s2) - hh1 \cdot s0 + atp \cdot chk \cdot (S - s0 - s2),$$

$$ds2/dt = - y0 \cdot s2 + atp \cdot z0 \cdot (S - s0 - s2) - hh2 \cdot s2 + hh1 \cdot (S - s0 - s2) - atp \cdot chk \cdot s2,$$

$$dr0/dt = atp \cdot z0 \cdot (R - r0 - r2) - r0 \cdot y0 - hh2 \cdot r0 + hh1 \cdot (R - r0 - r2) - atp \cdot chk \cdot r0,$$

$$dr2/dt = - atp \cdot z0 \cdot r2 + y0 \cdot (R - r0 - r2) + hh2 \cdot (R - r0 - r2) - hh1 \cdot r2 + atp \cdot chk \cdot (R - r0 - r2),$$

$$dq0/dt = y0 \cdot (Q - q0 - q2) - atp \cdot q0 \cdot z0 - hh2 \cdot q0 + hh1 \cdot (Q - q0 - q2),$$

$$dq2/dt = - y0 \cdot q2 + atp \cdot z0 \cdot (Q - q0 - q2) + hh2 \cdot (Q - q0 - q2) - hh1 \cdot q2,$$

$$dp0/dt = atp \cdot z0 \cdot (P - p0 - p2) - y0 \cdot p0 + hh2 \cdot (P - p0 - p2) - hh1 \cdot p0,$$

$$dp2/dt = - atp \cdot z0 \cdot p2 + y0 \cdot (P - p0 - p2) - hh2 \cdot p2 + hh1 \cdot (P - p0 - p2),$$

$$dy0/dt = q0 \cdot (Y - y0 - y2) - atp \cdot p0 \cdot y0 - hh2 \cdot y0 + hh1 \cdot (Y - y0 - y2),$$

$$dy2/dt = - atp \cdot z0 \cdot r2 + y0 \cdot (Y - y0 - y2) + hh2 \cdot (Y - y0 - y2) - hh1 \cdot y2$$

6. The Checkpoint-phosphatase General Kinase-Phosphatase (chp-GKP) system

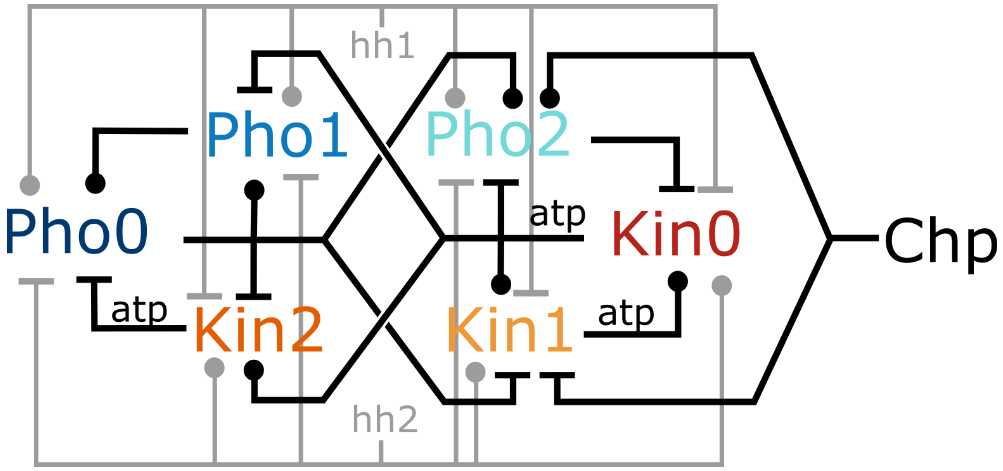

Figure S6. **GKP system with the interactions of the checkpoint phosphatase (chp-GKP system).** The checkpoint phosphatases Chp activates the phosphatase Pho2 and inhibits the kinase Kin1. The kinases (orange palette;, respectively in equations below) and phosphatases (blue palette;, respectively in equations below) are embedded in hysteretic harnesses (grey wiring). Ball-end edges show activation, dash-end edges show inhibition. *atp* labels in the edges specify the interactions that require a source of phosphate. Chp is a phosphatase, so it is not affected by the ATP/ADP ratio. **Initial conditions: Kin0**: z0 = 1.8, z2 = 1.2; **Kin1**: r0 = 1.8, r2 = 1.2; **Kin2**: p0 = 1.8, p2 = 1.2; **Pho0**: y0 = 1.2, y2 = 1.8; **Pho1**: q0 = 1.2, q2 = 1.8; **Pho2**: s0 = 1.2, s2 = 1.8.

$$dz0/dt = - s0 \cdot z0 + atp \cdot r0 \cdot (Z - z0 - z2) + hh2 \cdot (Z - z0 - z2) - hh1 \cdot z0,$$

$$dz2/dt = s0 \cdot (Z - z0 - z2) - atp \cdot r0 \cdot z2 - hh2 \cdot z2 + hh1 \cdot (Z - z0 - z2),$$

$$ds0/dt = y0 \cdot (S - s0 - s2) - atp \cdot z0 \cdot s0 + hh2 \cdot (S - s0 - s2) - hh1 \cdot s0 + Chp \cdot (S - s0 - s2),$$

$$ds2/dt = - y0 \cdot s2 + atp \cdot z0 \cdot (S - s0 - s2) - hh2 \cdot s2 + hh1 \cdot (S - s0 - s2) - Chp \cdot s2,$$

$$dr0/dt = atp \cdot z0 \cdot (R - r0 - r2) - r0 \cdot y0 - hh2 \cdot r0 + hh1 \cdot (R - r0 - r2) - Chp \cdot r0,$$

$$dr2/dt = atp \cdot z0 \cdot r2 + y0 \cdot (R - r0 - r2) + hh2 \cdot (R - r0 - r2) - hh1 \cdot r2 + Chp \cdot (R - r0 - r2),$$

$$dq0/dt = y0 \cdot (Q - q0 - q2) - atp \cdot q0 \cdot z0 - hh2 \cdot q0 + hh1 \cdot (Q - q0 - q2),$$

$$dq2/dt = - y0 \cdot q2 + atp \cdot z0 \cdot (Q - q0 - q2) + hh2 \cdot (Q - q0 - q2) - hh1 \cdot q2,$$

$$dp0/dt = atp \cdot z0 \cdot (P - p0 - p2) - y0 \cdot p0 + hh2 \cdot (P - p0 - p2) - hh1 \cdot p0,$$

$$dp2/dt = - atp \cdot z0 \cdot p2 + y0 \cdot (P - p0 - p2) - hh2 \cdot p2 + hh1 \cdot (P - p0 - p2),$$

$$dy0/dt = q0 \cdot (Y - y0 - y2) - atp \cdot p0 \cdot y0 - hh2 \cdot y0 + hh1 \cdot (Y - y0 - y2),$$

$$dy2/dt = - atp \cdot z0 \cdot r2 + y0 \cdot (Y - y0 - y2) + hh2 \cdot (Y - y0 - y2) - hh1 \cdot y2$$

7. Code availability

All quantitative descriptions of the models, as well as the parameters and state variables, are also accessible in the GitHub site of the project: rosherbal/thesis · GitHub

All graphics were generated using the code in rosherbal/thesis · GitHub in R [6] version 3.6, except for the panel e in figure 2 and b in figure 3, which were produced using oscill8 (http://oscill8.sourceforge.net/) and plotted using ggplot2 [7].

For the equations in sections 2-6, the conversions are: Cdk = z, PP2A = y, Cdc25 = r, Wee1 = s, PP1 = q, and Gwl = p.

8. Phylogenetic analysis

A 388-species eukaryotic genome dataset was collected (124 metazoa, 35 protist, 29 plant, 197 fungi, 2 slime mold, 1 Choanoflagellata) based on a previous study [8] and was supplemented by 20 archaeal and 9 bacterial genomes. Pairwise reciprocal best hit search was carried out by MMseqs2 (version: cc7d7da30ec779d6a2e886438f8295f59e2192f1-MPI)[9] using the following *Schizosaccharomyces pombe*, *Drosophila melanogaster* and *Mus Musculus* sequences as queries: *S. pombe*: Wee1 (SPCC18B5.03), Cdc25 (SPAC24H6.05), CDK (SPBC11B10.09, Cdc2 in *S. pombe*), Chk1 (SPCC1259.13, Rad27 in *S. pombe*), Chk2 (SPCC18B5.11c, Cds1 in *S. pombe*); *D. melanogaster*: Wee1 (P54350), Cdc25 (P20483), CDK (P23572), Chk1 (O61661) and Chk2 (O61267); *M. musculus*: Wee1 (P47810), Cdc25 (P48967), CDK (P11440), Chk1 (O35280) and Chk2 (Q9Z265). We used two other cell-cycle regulator proteins as positive and negative controls: Cks1 (SPBC1734.14c.1 in *S. pombe*, Q24152 in *D. melanogaster*, P61025 in *M. musculus*), NDR (SPCC417.06c.1 in *S. pombe*, Q9NBK5 in *D. melanogaster*, Q91VJ4 in *M. musculus*), respectively. A species was considered to have orthologs of these if reciprocal best hits were identified with at least two query sequences. Pairwise reciprocal best hit results were turned into a presence-absence matrix using R version 3.6.1 [6].

Single-copy gene family selection for species tree inference

BUSCO v3.0.2 together with orthodb v9 HMM profiles were used to tag and extract conserved and potentially single copy proteins from all species. Then, an all-vs-all MMSeqs search was carried out on the extracted proteins using the following parameters: -s 5.7, --max-seqs 1000000 -c 0.65 --cov-mode 0 -e 0001. HipMCL (version: e20476acc473) [10] was used to identify protein clusters within the protein similarity network using default prune, recovery and selection parameters together with the inflation parameter set to 1.5. Clusters considered to be phylogenetically informative were retained (containing at least 50 species representing at least 3 different phyla). Only single-copy clusters were used for the species tree inference. When a whole genome duplication prone species delegated more than one protein to a cluster, the closest single sequence was selected that displayed the largest average pairwise alignment score towards the single-copy members of the cluster. When gene duplication was observed from a species where whole genome duplication was not expected, the cluster was removed from further analysis. Selected protein clusters were manually ranked based on the taxa they contained representative protein from. Species tree was based on the 501 protein families. MAFFT with parameter "--auto" was used to yield multiple sequence alignments (MSAs). MSAs were then filtered with trimal (default, strict parameters), concatenated and a maximum likelihood tree was inferred in RAxML 8.1.2 [11] under the PROTGAMMAWAG model of evolution.

Correlated character evolution

To investigate the evolution of cell cycle regulators, we used discrete evolution models implemented in a Maximum Likelihood (ML) framework in BayesTraits V3.0.2[12] (http://www.evolution.rdg.ac.uk/BayesTraitsV3.0.2/BayesTraitsV3.0.2.html). By considering the proteins as traits, we can employ discrete models to test the correlation between them and its significance is established by the difference of the likelihoods of the independent and dependent models. Under the independent model, the two proteins evolve independently from each other and the model estimates four transition rates (the gain and loss rates for each trait). On the contrary, the dependent model estimates the transition rates for combinations of character states (presence-absence) that the two binary traits can simultaneously take and thus estimates eight transition rates (See more in Supplementary Table 2). Following Barker and Pagel [13], we constrained the gain rates ($0 \rightarrow 1$, independent model: alpha1 and alpha2 parameters, dependent model: q12, q13, q24, q34 parameters) to 0.02. This is the highest parameter value that constrains all genes to be gained once, which was shown to yield a biologically more realistic model and achieve better accuracy at detecting known functional links[13]. The number of ML attempts (mlt) was set to 25. We applied a likelihood ratio test (LRT) to compare the independent and dependent models' fit to the data based on their log-likelihood value. The likelihood ratio statistic is calculated as

LRT= 2[log-likelihood(better fitting model) – log-likelihood(worse fitting model)].

The likelihood ratio test is asymptotically distributed as a $\chi 2$ with degrees of freedom equalling 4 (critical values for $\chi 2$ at $p \leq 0.05$ at 4 degrees of freedom is 9.49, at $p \leq 0.001$ at 4 degrees of freedom is 18.47). The LRT was calculated from the mean of -log likelihood values per tree (See details in Supplementary Table 2). An LRT value <9.49 means no evidence for correlated

evolution. LRT values between 9.49 and 18.47 mean significant (p≤0.05), above 18.47 mean highly significant (p≤0.001) correlated evolution between the pair of proteins.

We inferred ancestral states for 22 nodes (see in Supplementary Table 2, Figure S7) by ancestral state reconstruction (ASR) using both the MultiState and the Discrete:Dependent model. For the ASR we fossilized the Root of the phylogenetic tree to state 0 and constrained the gain parameter rate values to 0.02 for the ASR Multistate, ASR Discrete:Dependent and Maximum Likelihood Ratio Test analysis as well.

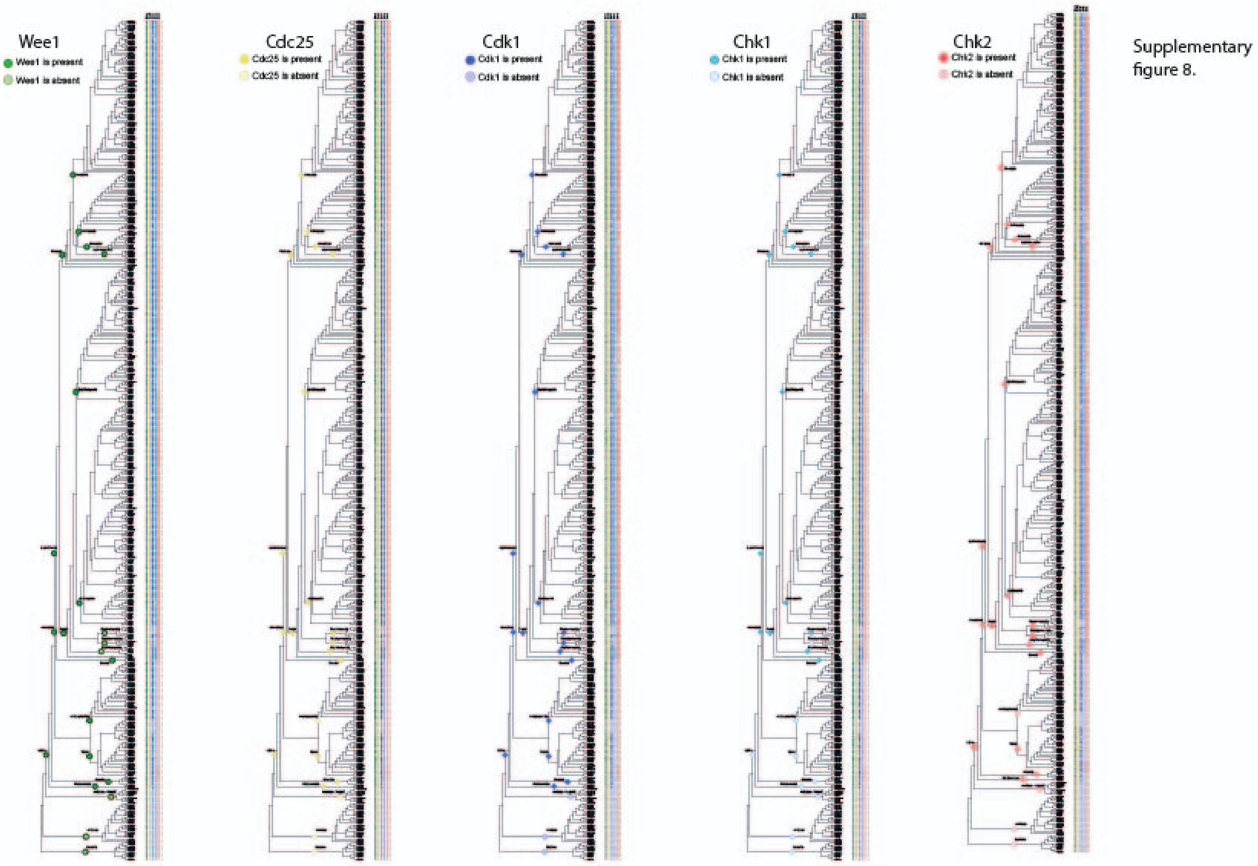

Figure S7. **Nodes with ancestral state reconstruction and the presence/absence of examined proteins across the 424-species tree** Ancestral state reconstruction (ASR) of 22 major nodes. We used PhyD3 [14] to illustrate the presence-absence matrix of Wee1, Cdc25, Cdk1, Chk1 and Chk2. See Supplementary table 2 for full taxon names of abbreviated names on phylogenetic trees. A high-resolution version of this figure is provided as a separate supplementary file. Readers can zoom in to read the details of that version.

Bibliography

1. Cardelli, L. Morphisms of reaction networks that couple structure to function. *BMC Syst. Biol.* **8**, 84 (2014).
2. Suwanmajo, T. & Krishnan, J. Mixed mechanisms of multi-site phosphorylation. *J. R. Soc.*

Interface **12**, (2015).
3. Salazar, C. & Höfer, T. Multisite protein phosphorylation--from molecular mechanisms to kinetic models. *FEBS J.* **276**, 3177–3198 (2009).
4. Qian, H. Phosphorylation energy hypothesis: open chemical systems and their biological functions. *Annu. Rev. Phys. Chem.* **58**, 113–142 (2007).
5. Wang, T. *et al.* Phosphorylation energy and nonlinear kinetics as key determinants for G2/M transition in fission yeast cell cycle. doi:10.1101/084400.
6. R Core Team. R: A Language and Environment for Statistical Computing. (2020).
7. Wickham, H. ggplot2: Elegant Graphics for Data Analysis. (2016).
8. Nagy, L. G., Merényi, Z., Hegedüs, B. & Bálint, B. Novel phylogenetic methods are needed for understanding gene function in the era of mega-scale genome sequencing. *Nucleic Acids Res.* **48**, 2209–2219 (2020).
9. Steinegger, M. & Söding, J. MMseqs2 enables sensitive protein sequence searching for the analysis of massive data sets. *Nat. Biotechnol.* **35**, 1026–1028 (2017).
10. Azad, A., Pavlopoulos, G. A., Ouzounis, C. A., Kyrpides, N. C. & Buluç, A. HipMCL: a high-performance parallel implementation of the Markov clustering algorithm for large-scale networks. *Nucleic Acids Res.* **46**, e33 (2018).
11. Stamatakis, A. RAxML version 8: a tool for phylogenetic analysis and post-analysis of large phylogenies. *Bioinformatics* **30**, 1312–1313 (2014).
12. Website. Meade, A., and Pagel, M. (2017). BayesTraits. http://www.evolution.rdg.ac.uk/BayesTraitsV3.0.1/BayesTraitsV3.0.1.html.
13. Barker, D., Meade, A. & Pagel, M. Constrained models of evolution lead to improved prediction of functional linkage from correlated gain and loss of genes. *Bioinformatics* **23**, 14–20 (2007).
14. Kreft, L., Botzki, A., Coppens, F., Vandepoele, K. & Van Bel, M. PhyD3: a phylogenetic tree viewer with extended phyloXML support for functional genomics data visualization. *Bioinformatics* **33**, 2946–2947 (2017).
